# Qualitätsindikatoren für die chirurgische Weiterbildung: Eine Analyse aktueller Konzepte und Umsetzungsmöglichkeiten des Perspektivforums Junge Chirurgie

**DOI:** 10.1007/s00104-026-02474-5

**Published:** 2026-03-03

**Authors:** Frederik Schlottmann, Joscha Mulorz, Benedikt Braun, Tobias Huber, Louisa Schuffert, Juliane Kröplin, Sabine Drossard, Sarah Lif Böhm, Maria E. Dey Hazra, Marit Herbolzheimer, Romina M. Rösch, Frederic Bouffleur, Stefanie Brunner, Josefine Schardey, Arash Motekallemi, Gerrit Freund, Manuela Oberlechner, Hruy Menghesha, Anna Lawson McLean, Thomas Schmitz-Rixen, Sebastian Schaaf

**Affiliations:** 1 Junges Forum der Deutschen Gesellschaft für Plastische, Rekonstruktive und Ästhetische Chirurgie (DGPRÄC), Klinik für Plastische, Ästhetische, Hand- und Wiederherstellungschirurgie, Medizinische Hochschule Hannover, Carl-Neuberg-Str. 1, 30625 Hannover, Deutschland; 2 Junges Forum der Deutschen Gesellschaft für Gefäßchirurgie und Gefäßmedizin (DGG), Klinik für Gefäß- und Endovaskularchirurgie, Universitätsklinikum Düsseldorf, Moorenstr. 5, 40225 Düsseldorf, Deutschland; 3 Themenreferat Nachwuchs, Berufsverband der Deutschen Chirurgie (BDC), Klinik für Unfall- und Wiederherstellungschirurgie an der Eberhard Karls Universität Tübingen, BG Klinik Tübingen, Schnarrenbergstr. 95, 72076 Tübingen, Deutschland; 4 Chirurgische Arbeitsgemeinschaft Junge Chirurgie (CAJC) der Deutschen Gesellschaft für Allgemein- und Viszeralchirurgie (DAV), Klinik für Allgemein‑, Viszeral- und Transplantationschirurgie, Universitätsmedizin Mainz, Langenbeckstr. 1, 55131 Mainz, Deutschland; 5 Arbeitskreis kinderchirurgischer Assistent:innen der Deutschen Gesellschaft für Kinder- und Jugendchirurgie (DGKJCH), Klinik und Poliklinik für Kinderchirurgie, Universitätsmedizin der Johannes Gutenberg-Universität Mainz, Langenbeckstr. 1, 55131 Mainz, Deutschland; 6 Perspektivforum Junge Chirurgie der Deutschen Gesellschaft für Chirurgie (DGCH), Klinik für Mund‑, Kiefer- und Plastische Gesichtschirurgie, Universitätsmedizin Rostock, Schillingallee 35, 18057 Rostock, Deutschland; 7 Perspektivforum Junge Chirurgie der Deutschen Gesellschaft für Chirurgie (DGCH), Klinik und Poliklinik für Allgemein‑, Viszeral‑, Transplantations‑, Gefäß- und Kinderchirurgie, Universitätsklinikum Würzburg, Oberdürrbacher Str. 6, 97080 Würzburg, Deutschland; 8 Junges Forum O und U der Deutschen Gesellschaft für Orthopädie und Unfallchirurgie (DGOU) und des Berufsverbands für Orthopädie und Unfallchirurgie (BVOU), Abteilung für Orthopädie und Unfallchirurgie, Städtisches Klinikum Karlsruhe, Moltkestr. 90, 76133 Karlsruhe, Deutschland; 9 Junges Forum O und U der Deutschen Gesellschaft für Orthopädie und Unfallchirurgie (DGOU) und des Berufsverbands für Orthopädie und Unfallchirurgie (BVOU), Orthopädische Privatpraxis Dr. Kuhlee, Hönower Str. 16, 12623 Berlin, Deutschland; 10 Junges Forum O und U der Deutschen Gesellschaft für Orthopädie und Unfallchirurgie (DGOU) und des Berufsverbands für Orthopädie und Unfallchirurgie (BVOU), BG Unfallklinik Murnau, Prof-Küntscher-Str. 8, 82418 Murnau am Staffelsee, Deutschland; 11 Junges Forum der Deutschen Gesellschaft für Thoraxchirurgie (DGT), Thoraxchirurgie, Thoraxklinik Heidelberg, Universitätsklinik Heidelberg, Röntgenstr. 1, 69126 Heidelberg, Deutschland; 12 Junges Forum der Deutschen Gesellschaft für Mund‑, Kiefer- und Gesichtschirurgie (DGMKG), Klinik und Poliklinik für Mund‑, Kiefer- und Gesichtschirurgie, Universitätsklinikum Heidelberg, Im Neuenheimer Feld 400, 69120 Heidelberg, Deutschland; 13 Chirurgische Arbeitsgemeinschaft Junge Chirurgie (CAJC) der Deutschen Gesellschaft für Allgemein- und Viszeralchirurgie (DGAV), Klinik und Poliklinik für Allgemein‑, Viszeral‑, Thorax- und Transplantationschirurgie, Universitätsklinikum Köln, Kerpener Str. 62, 50937 Köln, Deutschland; 14 Chirurgische Arbeitsgemeinschaft Junge Chirurgie (CAJC) der Deutschen Gesellschaft für Allgemein- und Viszeralchirurgie (DGAV), Klinik für Allgemein‑, Viszeral- und Transplantationschirurgie, LMU Klinikum, LMU München, Marchioninistr. 15, 81377 München, Deutschland; 15 Junges Forum der Deutschen Gesellschaft für Thorax‑, Herz- und Gefäßchirurgie (DGTHG), Klinik für Herzchirurgie, Klinikum Kassel, Mönchebergstr. 41–43, 34125 Kassel, Deutschland; 16 Vertreter der angestellten Fach- und Oberärzte der Deutschen Gesellschaft für Plastische, Rekonstruktive und Ästhetische Chirurgie (DGPRÄC), Klinik für Plastische, Rekonstruktive und Ästhetische Chirurgie – Handchirurgie und Schwerbrandverletztenzentrum, Universitätsklinikum Schleswig-Holstein, Ratzeburger Allee 160, 23538 Lübeck, Deutschland; 17 Vizepräsidentin Swiss Young Surgeons, Allgemein‑, Viszeral‑, Endokrin- und Transplantationschirurgie, HOCH Health Ostschweiz, Rorschacher Str. 95, 9000 St. Gallen, Schweiz; 18 Junges Forum der Deutschen Gesellschaft für Thoraxchirurgie (DGT), Klinik für Allgemein‑, Viszeral‑, Thorax- und Gefäßchirurgie, Universitätsklinikum Bonn, Venusberg-Campus 1, 53127 Bonn, Deutschland; 19 Klinik für Thoraxchirurgie, Helios Klinikum Bonn/Rhein-Sieg, Von-Hompesch-Str. 1, 53123 Bonn, Deutschland; 20 Junges Forum der Deutschen Gesellschaft für Neurochirurgie (DGNC), Klinik und Poliklinik für Neurochirurgie, Universitätsklinikum Jena, Am Klinikum 1, 07747 Jena, Deutschland; 21 Generalsekretär der Deutschen Gesellschaft für Chirurgie (DGCH), Luisenstr. 58/59, 10117 Berlin, Deutschland; 22 Perspektivforum Junge Chirurgie der Deutschen Gesellschaft für Chirurgie (DGCH) und Chirurgische Arbeitsgemeinschaft Junge Chirurgie (CAJC) der Deutschen Gesellschaft für Allgemein- und Viszeralchirurgie (DGAV), Klinik für Allgemein‑, Viszeral- und Thoraxchirurgie, Bundeswehrzentralkrankenhaus Koblenz, Rübenacher Str. 170, 56072 Koblenz, Deutschland

**Keywords:** Chirurgische Ausbildung, Qualitätsindikatoren im Gesundheitswesen, Gesundheitsreform, Chirurgische Verbundweiterbildung, Qualitätsbewertung, Surgical training, Quality indicators in the healthcare system, Healthcare reform, Surgical training alliances, Quality assessment

## Abstract

**Hintergrund:**

Die chirurgische Weiterbildung ist zentral für die Versorgungsqualität, wird jedoch in Deutschland kaum über valide Qualitätsindikatoren abgebildet, obwohl § 17b KHG eine qualitätsorientierte Finanzierung vorsieht.

**Ziel der Arbeit (Fragestellung):**

Die Arbeit verfolgt das Ziel, praxisnahe und messbare Qualitätsindikatoren zu entwickeln, die eine objektive Bewertung der Struktur‑, Prozess- und Ergebnisqualität der chirurgischen Weiterbildung ermöglichen.

**Material und Methoden:**

Zur Entwicklung der Indikatoren wurden gesetzliche Vorgaben analysiert, eine systematische Literaturrecherche zu „quality assessment surgical training“ wurde durchgeführt, und internationale Modelle wurden als Vergleich ausgewertet. Anschließend wurden zentrale Qualitätsdomänen identifiziert und im Expert:innenkonsens zu einem strukturierten Indikatorenset verdichtet.

**Ergebnisse:**

Es wird ein dreistufiges Indikatorenset vorgeschlagen: 1. Strukturindikatoren erfassen u. a. Curriculumqualität, Betreuungsverhältnis, Verbundweiterbildung und didaktische Qualifikation. 2. Prozessindikatoren umfassen strukturierte Weiterbildungsgespräche, operative Supervision einschließlich OPS-Kodierung für Weiterbildungseingriffe sowie Fortbildungsaktivität. 3. Ergebnisindikatoren betreffen Weiterbildungsdauer, Erfolgsquote der Facharztprüfung und standardisierte Zufriedenheit. Digitale Instrumente wie e‑Logbuch und Weiterbildungsregister ermöglichen erstmals valide, vergleichbare Daten.

**Diskussion:**

Standardisierte Qualitätsindikatoren schaffen Transparenz, erlauben Benchmarking und fördern eine lernende, datenbasierte Weiterbildungskultur. Eine jährliche digitale Erhebung sowie zentral koordinierte, anonymisierte Auswertungen sind essenziell, um Qualität zu sichern und zukünftige qualitätsorientierte Finanzierungsmodelle zu unterstützen.

## Abstract (en)

**Background:**

Advanced surgical training is essential for maintaining the quality of care; however, Germany still lacks validated quality indicators despite the legal requirements for quality-linked funding under §17b of the Hospital Funding Act.

**Objective:**

The aim of this work is to develop practical and measurable quality indicators that enable an objective assessment of the structural, procedural and outcome quality of advanced surgical training.

**Material and methods:**

To develop these indicators, legal frameworks were analyzed, a systematic literature search on quality assessment surgical training was conducted and international training models were analyzed as a comparison. Key quality domains were then identified and consolidated into a structured indicator set through expert consensus.

**Results:**

A three-stage set of indicators is proposed: 1) Structural indicators cover aspects such as curriculum quality, supervisor-trainee ratio, network-based training and teaching qualifications. 2) Process indicators include structured training discussions, operational supervision including surgical and procedural keys (OPS) coding for training interventions and continuing education activities. 3) Outcome indicators relating to the duration of training, the success rate of the specialist medical examination and standardized satisfaction. Digital tools, such as e‑logbooks and training registers enable valid and comparable data to be collated for the first time.

**Conclusion:**

Standardized quality indicators achieve transparency, enable benchmarking and foster a learning data-driven advanced training culture. Annual digital reporting and centralized anonymized assessments are essential for quality assurance and to support future quality-linked funding models.

## Hintergrund

Die chirurgische Weiterbildung bildet das Fundament für die Sicherstellung der Qualität und Zukunftsfähigkeit der chirurgischen Versorgung. Vor dem Hintergrund zunehmender Arbeitsverdichtung, fehlender Finanzierung, demografischer Veränderungen sowie hoher Anforderungen an Patientensicherheit und Effizienz besteht ein erhöhter Bedarf, die Qualität der chirurgischen Weiterbildung systematisch zu erfassen, zu vergleichen und zu verbessern [4, 5]. Bisher werden die Struktur- und Prozessparameter der ärztlichen Weiterbildung in Deutschland nicht systematisch erfasst. Dies ist jedoch notwendig und als Grundlage einer geplanten Finanzierung der Weiterbildung gesetzlich gefordert (Infobox 1).

### Infobox 1

Das Krankenhausstrukturgesetz (KHSG), das 2016 in Kraft trat, hat das Ziel, die Qualität der stationären Versorgung in Deutschland zu verbessern und gleichzeitig wirtschaftliche Anreize neu auszurichten. Es stellt einen zentralen Reformbaustein dar, da es erstmals die Finanzierung systematisch mit Aspekten der Versorgungsqualität verknüpft. Mit § 17b des Krankenhausfinanzierungsgesetzes (KHG) wurde die rechtliche Grundlage geschaffen, Qualitätsindikatoren als Bestandteil der Finanzierung der ärztlichen Weiterbildung einzuführen. Darin ist vorgesehen, dass bundesweit einheitliche und sektorübergreifende Qualitätsindikatoren vereinbart werden können, die auch für die ärztliche Weiterbildung relevant sind. Zudem ist geregelt, dass die maßgeblichen Vertragspartner (Spitzenverband Bund der Krankenkassen, Deutsche Krankenhausgesellschaft und Verband der Privaten Krankenversicherung) bis Ende 2025 auf Grundlage eines Konzepts des Instituts für das Entgeltsystem im Krankenhaus (InEK) entscheiden müssen, wie die mit der Weiterbildung verbundenen Mehrkosten sachgerecht finanziert werden. Vorgesehen sind Zuschläge oder Abschläge, die möglichst an Qualitätsindikatoren geknüpft und in Summe ausgeglichen sein sollen.

Diese Regelungen unterstreichen die strategische Ausrichtung des Gesetzgebers, die Qualität der ärztlichen Weiterbildung durch verbindliche und überprüfbare Indikatoren transparenter zu machen und über finanzielle Steuerungsinstrumente in die Versorgungsrealität zu integrieren. Dieser bereits 2016 formulierte Wunsch wird durch die Neuerungen im Rahmen des Krankenhausversorgungsverbesserungsgesetzes (KHVVG), welches am 01.01.2025 in Kraft trat, mehr denn je zur Realität, da bisher keine Qualitätsindikatoren zur Bewertung der ärztlichen, insbesondere der chirurgischen Weiterbildung, etabliert wurden.

In einem ersten Schritt müssen nun geeignete Qualitätsindikatoren identifiziert und klar definiert werden, die als standardisiertes, valides und vergleichbares Instrumentarium eine objektive Mess- und Vergleichbarkeit ermöglichen. Das vorliegende Positionspapier formuliert einen Vorschlag für konkrete, praxisnahe sowie messbare Qualitätsindikatoren für die chirurgische Weiterbildung. Nach Etablierung können diese Transparenz schaffen, Stärken und Schwächen identifizieren und so die Weiterbildungsstrukturen nachhaltig verbessern.

## Zielsetzung und konzeptionelle Grundlagen

Die vorgeschlagenen Qualitätsindikatoren sollen:

- die Qualität der chirurgischen Weiterbildung objektivierbar machen und diese nachhaltig sichern,
- als Grundlage für interne und externe Evaluationen dienen und somit vergleichbare Standards zwischen Weiterbildungsstätten etablieren,
- zur kontinuierlichen Verbesserung der Weiterbildungsstrukturen beitragen.

Nebeneffekte könnten zugleich die Steigerung der Attraktivität des chirurgischen Berufsbildes und die Ermöglichung eines administrativ vereinfachten Wechsels (Standardisierung) zwischen Weiterbildungsstätten im Rahmen von Verbundweiterbildungen sein.

Dabei orientieren sich die Indikatoren an folgenden Prinzipien:

- *Relevanz:* Berücksichtigung der klinischen, bildungspolitischen und berufspraktischen Bedeutung,
- *Validität:* Abbildung zentraler und inhaltlich bedeutsamer Aspekte der chirurgischen Weiterbildung,
- *Reliabilität:* Gewährleistung einer konsistenten und reproduzierbaren Messung,
- *Praktikabilität:* Sicherstellung einer ressourcenschonenden, klar strukturierten und anwenderfreundlichen Erhebung,
- *Vergleichbarkeit:* Ermöglichung belastbarer Vergleiche zwischen Institutionen sowie im zeitlichen Verlauf.

Nach Ansicht des Autorenteams steht zunächst die systematische Erfassung aller Chirurginnen und Chirurgen in Weiterbildung im Vordergrund. Nur so können der Verlauf der Weiterbildung, Wechsel zwischen Weiterbildungsstätten oder auch Abbrüche nachvollzogen werden. Zwar führen einige wenige Landesärztekammern (LÄK), beispielsweise in Hessen, spezifische Statistiken, die überwiegende Anzahl tritt jedoch erst zum Zeitpunkt der Anmeldung zur Facharztprüfung in direkten Kontakt mit den Weiterzubildenden [1]. Eine jährliche Meldung des Weiterbildungsfortschritts (z. B. OP-Katalog) an die zuständige LÄK wäre daher ein wichtiger Schritt, um Transparenz und Steuerbarkeit zu verbessern.

Mit dem e‑Logbuch bestehen bereits geeignete Strukturen für eine zentrale Erfassung, deren Potenzial jedoch noch nicht vollständig genutzt wird. Die Einführung eines Weiterbildungsregisters in Bayern ab Oktober 2025 stellt somit einen Schritt in die richtige Richtung dar – hin zu einer systematischen Erhebung, einer verbesserten Nachvollziehbarkeit des Weiterbildungsverlaufs und einer vereinfachten Koordination zwischen Weiterbildungsstätten und LÄK.

## Systematik der Qualitätsindikatoren

Zur systematischen Erfassung und Bewertung der Weiterbildungsqualität werden die vorgeschlagenen Indikatoren in 3 Kategorien unterteilt. Diese orientieren sich an etablierten Qualitätsdimensionen und ermöglichen eine differenzierte Betrachtung der Rahmenbedingungen, Abläufe und Ergebnisse der chirurgischen Weiterbildung.

Die Indikatoren gliedern sich in 3 Kategorien:

- *Strukturindikatoren:* Rahmenbedingungen der Weiterbildung,
- *Prozessindikatoren:* Abläufe und Tätigkeiten in der Weiterbildung,
- *Ergebnisindikatoren:* erreichte Kompetenzen und Zufriedenheit.

## Strukturindikatoren

### 1. Verfügbarkeit eines strukturierten Weiterbildungscurriculums

- *Definition:* Wird zu Beginn der Weiterbildung ein strukturiertes Weiterbildungscurriculum ausgehändigt, das digital dokumentiert wird und sich an der aktuellen Weiterbildungsordnung (WBO) orientiert?
- *Zielwert:* 100 %
- *Datenquelle:* Unterlagen bei der Einreichung bei der zuständigen LÄK zur Beantragung einer Weiterbildungsermächtigung, e‑Logbuch

Derzeit ist unklar, wie viele Ärztinnen und Ärzte zu Beginn ihrer Weiterbildung das Curriculum tatsächlich kennen. Ein strukturiertes, klinikspezifisches Curriculum ist jedoch Voraussetzung für die Weiterbildungsermächtigung und sollte die Umsetzung kompetenzbasierter Lernziele transparent festlegen und öffentlich einsehbar sein. Ein von den chirurgischen Fachgesellschaften entwickeltes Muster-Curriculum, das standortbezogen angepasst und regelmäßig aktualisiert wird, wäre sinnvoll.

Ein qualitativ hochwertiges Curriculum sollte auf einem bundesweit einheitlichen, kompetenzbasierten Rahmen basieren, der klare Lernziele, Rotationen und praxisnahe Trainingsmethoden (z. B. Simulationen, OP-Kurse) definiert. Regelmäßige Evaluationen, didaktische Begleitung und Supervision sichern dabei die Qualität der Weiterbildung und die Patientensicherheit.

### 2. Betreuungsverhältnis fachärztlicher Mentor:innen zu Weiterzubildende

- *Definition:* durchschnittliches Verhältnis zur Sicherstellung individueller Betreuung
- *Zielwert:* 1 FA/FÄ mit entsprechender didaktischer Qualifikation (zertifizierter Train-the-Trainer-Kurs) zu 3 Weiterzubildenden
- *Datenquelle:* Personalstatistik, Abteilungsbefragung, Selbstauskunft

Nicht jede Fachärztin bzw. jeder Facharzt ist automatisch zur Weiterbildung berechtigt – hierfür ist eine offizielle Weiterbildungsermächtigung erforderlich. Daher wäre es sinnvoll, dass entsprechend didaktisch qualifizierte Fachärzt:innen als Mentor:innen in der Weiterbildung fungieren könnten. Im Zuständigkeitsbereich der LÄK Berlin besteht bereits ein Mentoring-System, bei dem ab 5 Weiterzubildenden Mentor:innen benannt werden müssen, die in die Vermittlung und Evaluation der Inhalte – etwa durch Abzeichnung des e‑Logbuchs oder jährliche Weiterbildungsgespräche – eingebunden sind. Langfristig könnte auch eine Orientierung an den Facharztzahlen der jeweiligen Leistungsgruppen nach KHVVG erfolgen (3 Fachärzte = max. 9 Weiterzubildende).

### 3. Teilnahme an strukturierten Weiterbildungsverbünden

- *Definition:* Anteil chirurgischer Weiterbildungsassistent:innen, die an einem formalisierten Weiterbildungsverbund teilnehmen, wenn die jeweilige Weiterbildungsstätte keine vollumfängliche Weiterbildungsermächtigung hat
- *Zielwert:* beginnend ab 2026: ≥ 50 % bis 2032 (mittelfristig ≥ 80 % bis 2038): Teilnahme ja/nein
- Datenquelle: Selbstauskunft, Kooperationsverträge, e‑Logbuch

Die Verbundweiterbildung stellt ein strukturiertes Kooperationsmodell dar, das ambulante und stationäre Weiterbildungseinrichtungen systematisch vernetzt, um eine umfassende und qualitativ hochwertige Abdeckung der curricularen Inhalte sicherzustellen [3]. Sie trägt zugleich zur nachhaltigen Verbesserung der Arbeits- und Ausbildungsbedingungen bei. Gerade in der Chirurgie, die durch hohe Spezialisierung, komplexe klinische Anforderungen und die fortschreitende Ambulantisierung geprägt ist, gewinnen intersektorale Weiterbildungsverbünde zunehmend an Bedeutung [6]. Diese Entwicklung wird durch die Einführung von Leistungsgruppen mit spezifischen Strukturvorgaben zusätzlich begünstigt. Wie ein Positionspapier der Jungen Foren der chirurgischen Fachgesellschaften betont, ist die Etablierung solcher Verbünde „essentiell, um die chirurgische Versorgung und Weiterbildung zu sichern“ [5]. Ein qualitativ hochwertiger Weiterbildungsverbund sollte über strukturierte Rotationspläne, trägerübergreifende Verträge, ein standardisiertes Curriculum sowie didaktisch qualifizierte Weiterbildende verfügen [5]. Erfahrungen aus Pilotprojekten – etwa Rotationen in medizinische Versorgungszentren – zeigen positive Effekte auf praktische Kompetenzentwicklung, Motivation und Arbeitszufriedenheit [6]. Verbundbasierte Modelle ermöglichen zudem die sinnvolle Verbindung von Ambulantisierung und Zentralisierung und sichern so die Kontinuität chirurgischer Weiterbildung trotz zunehmend fragmentierter Versorgungsstrukturen. Die Verantwortung für die Organisation solcher Verbünde darf jedoch nicht allein bei den Weiterzubildenden liegen, sondern bei den Weiterbildungsermächtigten und den zuständigen Landesärztekammern. Erforderlich sind klare politische und strukturelle Rahmenbedingungen, eine verlässliche Finanzierung sowie die Einbindung der Fachgesellschaften [4].

### 4. Didaktische Qualifizierung der Weiterbildungsermächtigten

- *Definition:* Qualifizierung im Rahmen eines Train-the-Trainer-Kurses, durch hochschuldidaktische Kurse oder vergleichbare Qualifikationen (Master of Medical Education o. Ä.)
- Identische Qualifizierung auch der FA/FÄ (Mentor:innen), die in die Weiterbildung eingebunden werden
- *Zielwert:* 100 %
- *Datenquelle:* Selbstauskunft, Zertifikatsnachweise, e‑Logbuch

Erfreulicherweise ist eine Ausweitung der Kursangebote zu beobachten: So bietet die Landesärztekammer Nordrhein jährlich rund 90 Kurse an, und auch die Deutsche Gesellschaft für Chirurgie organisiert mehrfach im Jahr entsprechende Kursformate. Perspektivisch wäre eine Multiplikatorfunktion denkbar, bei der bereits Qualifizierte abteilungsinterne Schulungen anbieten.

Neben der didaktischen Ausbildung gewinnt die Vermittlung von Führungskompetenzen an Bedeutung. Weiterbildungsermächtigte übernehmen nicht nur Lehr-, sondern auch Führungsaufgaben und sollten Kenntnisse in Mitarbeiterführung, Kommunikation, Konfliktmanagement und Organisationsentwicklung mitbringen. Aufbauprogramme mit Elementen aus Management- oder MBA-Curricula könnten hier eine sinnvolle Ergänzung bestehender Qualifikationsmodelle darstellen.

## Prozessindikatoren

### 1. Durchführung strukturierter Weiterbildungsgespräche

- *Definition:* Anteil der Weiterbildungsassistent:innen mit dokumentierten Jahresgesprächen
- *Zielwert:* 100 %
- *Datenquelle:* Personalakte, Vordruck LÄK/e-Logbuch

Strukturierte Weiterbildungsgespräche sind zentral für eine qualitativ hochwertige chirurgische Weiterbildung, ihre Umsetzung ist jedoch oft durch Ausfälle, hohe Dienstbelastung oder Personalwechsel erschwert. Dennoch sollte eine konsequente und qualitativ hochwertige Durchführung sichergestellt werden. Entscheidend ist, dass die Gespräche nicht nur formal, sondern inhaltlich substanziell geführt werden. Eine verbindliche Verankerung im Curriculum sowie die Integration in nutzerfreundliche Systeme wie das e‑Logbuch können die Umsetzung und Dokumentation erleichtern.

### 2. Operative Supervision

- *Definition:* Anteil durchgeführter Eingriffe durch Weiterbildungsassistent:innen im Verhältnis zur Gesamtanzahl der durchgeführten weiterbildungsrelevanten Eingriffe
- *Zielwert:* ≥ 80 %
- *Datenquelle:* OP-Dokumentation, e‑Logbuch

In Deutschland fehlen bislang valide Daten zur Anzahl tatsächlich durchgeführter Weiterbildungseingriffe. Ein wichtiger Schritt zur besseren Bewertung der operativen Weiterbildung ist daher die Einführung eines eigenen Operationen- und Prozedurenschlüssels (OPS-Codes) für „Weiterbildungseingriffe“, für den die Deutsche Gesellschaft für Chirurgie (DGCH) 2025 einen Antrag beim Bundesinstitut für Arzneimittel und Medizinprodukte (BfArM) gestellt hat. Der Code soll den zeitlichen Mehraufwand und die operative Supervision – etwa Eingriffe unter Anleitung – abbilden.

Damit ließen sich erstmals bundesweit belastbare Daten zu Umfang und Struktur der operativen Weiterbildung erheben, was eine objektivere Bewertung von Qualität und Quantität sowie gesundheitspolitische Analysen zu regionalen Unterschieden ermöglichen würde. Langfristig könnte dies auch die Grundlage für ein Vergütungsmodell des didaktischen Mehraufwands schaffen.

Gleichzeitig hat diese vorgeschlagene OPS-Codierung Grenzen: Eine rein zeitbasierte Erfassung spiegelt nicht notwendigerweise die Qualität der Supervision wider und kann Fehlanreize erzeugen. Viele didaktische Leistungen – etwa Vorbereitung oder Nachbesprechung – werden zudem nicht erfasst. Daher sollte die OPS-Codierung stets durch qualitative Indikatoren ergänzt und in ein strukturiertes Weiterbildungskonzept eingebettet werden.

### 3. Teilnahme an Fortbildungsveranstaltungen

- *Definition:* durchschnittliche Anzahl besuchter chirurgischer Fortbildungen pro Jahr (beispielsweise OP-Kurs, Simulationsszenarien, Hands-on-Kurse, Seminare, Kongresse)
- *Zielwert:* ≥ 3 Veranstaltungen pro Jahr (orientierend an Fortbildungspunkten für FÄ)
- *Datenquelle:* Teilnahmebescheinigungen, interne Dokumentation, EFN-Nummer/Weiterbildungskonto bei der LÄK

Zur Sicherung der Weiterbildungsqualität ist die regelmäßige Teilnahme aller Assistenzärzt:innen an fachlich relevanten Fortbildungen unerlässlich. Dazu gehören theoretische Formate (Seminare, Kongresse, Webinare) ebenso wie praktische Trainings (OP-Kurse, Workshops, Simulationen), die getrennt erfasst und bewertet werden sollten. Auch abteilungsinterne Fortbildungen, etwa in Frühbesprechungen oder strukturierten Teaching-Terminen, tragen wesentlich zu einer gelebten Weiterbildungskultur bei.

Orientiert an den Vorgaben der Landesärztekammern sollten innerhalb von 5 Jahren 250 Fortbildungspunkte (∅ 50 Punkte/Jahr) erzielt werden, etwa durch die Teilnahme an mindestens 3 Fortbildungsveranstaltungen jährlich, ergänzt durch interne Angebote oder akkreditierte Kurse von Fachgesellschaften. Dafür sind angemessene zeitliche und finanzielle Ressourcen wie Fortbildungstage und individuelle Budgets erforderlich. In strukturschwächeren Regionen können digitale Formate und regionale Verbundlösungen unterstützen. Langfristig könnte die Fortbildungsteilnahme als messbarer Indikator für Weiterbildungsqualität dienen und in Evaluationen oder Fördermodelle einfließen.

## Ergebnisindikatoren

### 1. Dauer der Weiterbildung

- *Definition:* systematische Erhebung und Dokumentation der tatsächlichen Weiterbildungsdauer (inklusive Rotationen, Teilzeitmodelle, Fehlzeiten)
- *Zielwert:* ≥ 85 % Anteil der Facharztanerkennungen innerhalb der Regelweiterbildungszeit (unter Berücksichtigung von anerkannten Verzögerungen (z. B. Forschungsaufenthalte, Elternzeit etc.))
- Rückmeldungssystem an Weiterbildungsermächtigte bei systematischer Überschreitung der Weiterbildungszeit inklusive strukturierter Abfrage der Ursachen
- *Datenquelle*: e‑Logbuch und Facharztanmeldung bei der LÄK

Die Weiterbildungsdauer ist ein zentraler Indikator für die Effektivität und Strukturqualität chirurgischer Programme [1]. Der Anteil der in Regelzeit abgeschlossenen Facharztanerkennungen sollte jährlich erfasst und anonymisiert veröffentlicht werden, etwa über Weiterbildungsstatistiken der Ärztekammern. Ein Frühwarnsystem könnte strukturelle Defizite frühzeitig identifizieren, indem Weiterbildungsstätten bei wiederholten Überschreitungen der Regelzeit automatisiert informiert werden. Durch strukturierte Rückmeldungen mit Angabe möglicher Gründe (z. B. Teilzeit, Elternzeit, Auslandsaufenthalte) lassen sich individuelle von systemischen Ursachen unterscheiden.

Ziel ist mehr Transparenz und Planbarkeit – nicht Sanktionierung, sondern die Förderung einer effizienten und fairen Weiterbildung. Mittelfristig könnten diese Daten in Förderkriterien oder Qualitätssiegel einfließen.

### 2. Erfolgsquote Facharztprüfung

- *Definition:* Anteil bestandener Facharztprüfungen beim ersten Versuch nach Absolvierung der Regelweiterbildungszeit
- *Zielwert:* ≥ 90 %
- *Datenquelle:* LÄK, klinikinterne Erhebung

Die Erfolgsquote bei der Facharztprüfung ist ein zentraler Indikator der Weiterbildungsqualität. Besonders aussagekräftig ist der Anteil bestandener Prüfungen beim ersten Versuch nach Regelweiterbildungszeit; eine Zielquote von ≥ 90 % gilt als Benchmark für strukturierte und kompetenzorientierte Weiterbildung.

Zur Qualitätssicherung sollten Beginn, Anmeldung, Freigabe und Prüfungstermin standardisiert dokumentiert und ausgewertet werden, wobei Bearbeitungszeiten der Kammern separat auszuweisen sind. Die Verantwortung der Weiterbildungsstätten unterstützt durch die Fachgesellschaften umfasst auch die gezielte Prüfungsvorbereitung und Kompetenzentwicklung. Standardisierte Zwischenprüfungen können helfen, Wissenslücken frühzeitig zu erkennen. Eine transparente Veröffentlichung aggregierter Erfolgsdaten pro Weiterbildungsstätte würde langfristig Qualitätsentwicklung fördern und positive Anreize für strukturierte Weiterbildungskonzepte schaffen.

### 3. Zufriedenheit der Weiterbildungsassistent:innen und Weiterbildungsermächtigten

- *Definition:* durchschnittliche Zufriedenheit im Rahmen eines standardisierten Fragebogens durchgeführt durch die Fachgesellschaften
- *Zielwert:* ≥ 80 %
- *Datenquelle:* jährliche anonyme Befragung der Weiterbildungsassistent:innen und Weiterbildungsermächtigten

Die Zufriedenheit von Weiterbildungsassistent:innen und Weiterbildungsermächtigten ist ein zentraler Indikator der Weiterbildungsqualität und sollte regelmäßig anonym auf einem standardisierten, noch zu entwickelnden Erhebungsbogen, welcher die Qualitätskriterien abbildet, erfasst werden. Zielwert ist eine durchschnittliche Zufriedenheit von mindestens 80 %. Die Erhebung sollte digital, zentral koordiniert und idealerweise durch die Fachgesellschaften durchgeführt werden, wobei die Landesärztekammern eng eingebunden werden sollten. Zwar existieren bereits kommerzielle Karriere- und Bewertungsportale, deren Datennutzung in anderen Branchen jedoch erhebliche Bias-Risiken zeigt – etwa durch Selbstselektion, Extrembewertungen und potenzielle Interessenkonflikte infolge werbebasierter Geschäftsmodelle. Daher sollte eine unabhängige, wissenschaftlich validierte Plattform zur standardisierten Zufriedenheitserhebung etabliert werden. Ein jährliches Monitoring würde Trends sichtbar machen, gezielte Verbesserungsmaßnahmen ermöglichen und langfristig als Grundlage für Zertifizierungen oder Förderprogramme dienen.

*Das Autorenteam schlägt folgende konkrete Qualitätsindikatoren zur systematischen und standardisierten Erfassung der Weiterbildungsqualität in Deutschland vor (*Infobox 2*).*

## Der Blick über den Tellerrand

Ein Blick über die nationalen Grenzen hinaus zeigt, dass zahlreiche Länder bereits strukturierte und evidenzbasierte Systeme zur Bewertung und Weiterentwicklung der chirurgischen Weiterbildung implementiert haben – und damit wertvolle Anhaltspunkte für die nationale Diskussion liefern (Tab. 1).

**Tab. 1:** Übersicht der Weiterbildungs- und Evaluationssysteme im internationalen Vergleich

Land	Curriculum & Struktur	Evaluation & Dokumentation	Indikatoren/Assessments	Besonderheiten
Vereinigtes Königreich (UK)	Intercollegiate Surgical Curriculum Programme (ISCP); national einheitlich	Elektronisches Logbuch, ARCP (Annual Review of Competency Progression)	Workplace-Based Assessments (WBAs), Multi-Source-Feedback, operative Fallzahlen, Teaching	Pflicht zur jährlichen Kompetenzprüfung; zentrale digitale Infrastruktur
Irland	Surgical Training Committee (STC), Royal College of Surgeons (RCSI)	„Professional Competence Scheme“ mit jährlicher peer-reviewter Aktivitätsdokumentation	Kurse, OP-Zahlen, Lehre, Forschung; Zufriedenheitsbefragungen	Verpflichtende Fortbildungs- und Qualitätsnachweise pro Jahr
Niederlande	CanMEDS-basierte Weiterbildung mit EPAs	Zentral elektronisches Portfolio; regelmäßige Peer-Evaluation	EPAs, Feedbackkultur, strukturierte Supervision, operative Kompetenz	Kombination aus standardisierten Modulen, Feedbackprozessen und Outcome-Bewertung
USA	ACGME Accreditation mit SCORE®-Curriculum in > 95 % der chirurgischen Programme	Webbasierte Lernplattformen; Monitoring über operative Logbücher & Assessment-Plattformen	Milestones, EPAs, operative Fallzahlen; NSQIP-Daten (Qualität), Prüfungserfolg	Starke Verknüpfung von Weiterbildung und Versorgungsqualität durch externe Qualitätserhebungen (z. B. ACS NSQIP)
Schweiz	SIWF/ISFM-Weiterbildungsprogramm; 6‑jährige Facharztweiterbildung; Core Surgical Curriculum als gemeinsame Basis	Elektronisches Logbuch/OP-Katalog; jährliches Core Surgical Exam; verpflichtende AbA-Dokumentation	Mini-CEX/DOPS (≥ 4/Jahr), EPAs, operative Fallzahlen, Kongress‑/Kursnachweise	Starke nationale Steuerung durch SIWF; schrittweise Umstellung auf kompetenzbasierte Weiterbildung mit EPA-Tools und zentral harmonisierter Grundweiterbildung (CSC)
Deutschland (Zielmodell)	Weiterbildungsordnung der LÄK; Positionspapier Junge Chirurgie (Qualitätsindikatoren)	e‑Logbuch in Vorbereitung/Einführung, aber noch uneinheitlich	OP-Supervision, Feedbackgespräche, Fortbildung, Zufriedenheit, Wissenschaftlichkeit	Ziel: bundesweite Erhebung qualitätsgesicherter Indikatoren zur Steuerung und Finanzierung der Weiterbildung

Eine systematische PubMed-Recherche (Suchbegriff: *„quality assessment surgical training“ und verwandte MeSH-Terms*, 15.199 Treffer, Juli 2025) identifizierte internationale Ansätze zur Bewertung und Steuerung der chirurgischen Weiterbildungsqualität. Ziel war die Analyse vorhandener Evidenz und Good-Practice-Beispiele sowie deren potenzielle Übertragbarkeit auf das deutsche System.

Ein Delphi-Konsensus-Paper (2024) definierte zentrale Bewertungsdomänen – darunter OP-Zahlen, Vielfalt, Supervision und Feedback – und lieferte methodische Impulse zur Entwicklung valider Indikatoren [8]. Ein systematisches Review (2024) mit 68 Studien nannte OP-Volumen, arbeitsplatzbasierte Assessments, Feedback und wissenschaftliche Aktivitäten als häufige, jedoch teils unvalidierte Indikatoren [2]; weitere Reviews betonen die Notwendigkeit empirischer Validierung und internationaler Vergleichbarkeit [7].

Internationale Programme verdeutlichen die Machbarkeit solcher Systeme: Das britische *Intercollegiate Surgical Curriculum Programme (ISCP)* nutzt digitale Logbücher und regelmäßige Assessments; Irland und die Niederlande arbeiten mit kompetenzbasierten Frameworks und Peer-Evaluationen; in den USA verknüpfen Entrustable Professional Activities (EPAs) und nationale Register (z. B. SCORE®, ACS NSQIP) Weiterbildung und Outcome-Daten.

Diese Beispiele zeigen, dass digitale Dokumentation, klare Kompetenzziele, regelmäßige Assessments und Benchmarking zentrale Erfolgsfaktoren sind – und bieten zugleich eine erprobte Grundlage für eine transparente, kompetenz- und outcomeorientierte Weiterbildungskultur in Deutschland.

### Infobox 2

Vom Autorenteam vorgeschlagene konkrete Qualitätsindikatoren zur systematischen und standardisierten Erfassung der Weiterbildungsqualität in Deutschland

1. Strukturindikatoren
  Verfügbarkeit eines strukturierten Weiterbildungscurriculums
  Betreuungsverhältnis fachärztlicher Mentor:innen zu Weiterzubildende
  Teilnahme an strukturierten Weiterbildungsverbünden
  Didaktische Qualifizierung der Weiterbildungsermächtigten
2. Prozessindikatoren
  Durchführung strukturierter Weiterbildungsgespräche
  Operative Supervision
  Teilnahme an Fortbildungsveranstaltungen
3. Ergebnisindikatoren
  Dauer der Weiterbildung
  Erfolgsquote Facharztprüfung
  Zufriedenheit der Weiterbildungsassistent:innen und Weiterbildungsermächtigten

Diese Maßnahmen bilden die Grundlage für eine moderne, transparente und zukunftsfähige Weiterbildungskultur in der Chirurgie – mit dem Ziel, Qualität, Attraktivität und Verbindlichkeit der chirurgischen Weiterbildung langfristig zu stärken.

## Fazit für die Praxis

Die Einführung und nachhaltige Verankerung von Qualitätsindikatoren in der chirurgischen Weiterbildung sind ein zentraler Schritt zur Sicherung von Versorgungsqualität und Nachwuchsförderung. Ziel ist nicht Kontrolle, sondern der Aufbau einer datengestützten, lernenden Weiterbildungsstruktur, die Transparenz schafft, Stärken sichtbar macht und gezielt auf Verbesserungsbedarfe reagiert. Die Erhebung der Indikatoren sollte jährlich auf Abteilungsebene erfolgen, in bestehende Qualitätssicherungsprozesse integriert und von Fachgesellschaften, Landesärztekammern und Weiterbildungsverbünden unterstützt werden. Eine digital gestützte, zentral koordinierte Auswertung ermöglicht valide, vergleichbare Daten und reduziert den Aufwand vor Ort. Ein anonymisiertes Benchmarking kann Abteilungen gezielte Rückmeldung geben und zugleich übergreifende Entwicklungen sichtbar machen. Die vorgeschlagenen, praxisnah und konsensorientiert entwickelten Indikatoren schaffen die Grundlage, Weiterbildung messbar, steuerbar und perspektivisch auch finanzierbar zu gestalten. Durch § 17b KHG bestehen hierfür bereits rechtliche Rahmenbedingungen – das anstehende Konzept der Vertragsparteien bis Ende 2025 bietet die Chance, Qualitätsindikatoren als Steuerungsinstrument verbindlich zu etablieren.
